# A Computational Model to Investigate Astrocytic Glutamate Uptake Influence on Synaptic Transmission and Neuronal Spiking

**DOI:** 10.3389/fncom.2012.00070

**Published:** 2012-10-01

**Authors:** Sushmita L. Allam, Viviane S. Ghaderi, Jean-Marie C. Bouteiller, Arnaud Legendre, Nicolas Ambert, Renaud Greget, Serge Bischoff, Michel Baudry, Theodore W. Berger

**Affiliations:** ^1^Department of Biomedical Engineering, University of Southern CaliforniaLos Angeles, CA, USA; ^2^Department of Electrical Engineering, University of Southern CaliforniaLos Angeles, CA, USA; ^3^Neuroscience Program, University of Southern CaliforniaLos Angeles, CA, USA; ^4^Rhenovia PharmaMulhouse, France; ^5^Graduate College of Biomedical Sciences, Western University of Health SciencesPomona, CA, USA

**Keywords:** astrocyte, glutamate uptake, glutamatergic synapse, computational model, neuron spiking

## Abstract

Over the past decades, our view of astrocytes has switched from passive support cells to active processing elements in the brain. The current view is that astrocytes shape neuronal communication and also play an important role in many neurodegenerative diseases. Despite the growing awareness of the importance of astrocytes, the exact mechanisms underlying neuron-astrocyte communication and the physiological consequences of astrocytic-neuronal interactions remain largely unclear. In this work, we define a modeling framework that will permit to address unanswered questions regarding the role of astrocytes. Our computational model of a detailed glutamatergic synapse facilitates the analysis of neural system responses to various stimuli and conditions that are otherwise difficult to obtain experimentally, in particular the readouts at the sub-cellular level. In this paper, we extend a detailed glutamatergic synaptic model, to include astrocytic glutamate transporters. We demonstrate how these glial transporters, responsible for the majority of glutamate uptake, modulate synaptic transmission mediated by ionotropic AMPA and NMDA receptors at glutamatergic synapses. Furthermore, we investigate how these local signaling effects at the synaptic level are translated into varying spatio-temporal patterns of neuron firing. Paired pulse stimulation results reveal that the effect of astrocytic glutamate uptake is more apparent when the input inter-spike interval is sufficiently long to allow the receptors to recover from desensitization. These results suggest an important functional role of astrocytes in spike timing dependent processes and demand further investigation of the molecular basis of certain neurological diseases specifically related to alterations in astrocytic glutamate uptake, such as epilepsy.

## Introduction

Until a few decades ago the quest to better understand high level brain functions, such as learning, memory, and cognition, mainly focused on investigating the rapid, spike-based information processing performed by neurons. Glial cells, and among them astrocytes, were largely regarded as passive support cells, providing neurons with nutrition and structural support without directly participating in information processing functions (Kandel et al., 1991). Over the past 20 years, however, a growing body of evidence has demonstrated that astrocytes do participate in bi-directional signaling with neurons and, therefore, possibly play an important role in shaping communication in the brain (Volterra and Steinhauser, 2004; Perea and Araque, 2005). These findings demand a revision of the traditional neuron-centric model used to explain higher order brain functions to include astrocytes as part of a neuron-glia network model. Within this new framework, signaling includes both, fast spike-based processing and slower modulation mediated by astrocytic elements (Nedergaard and Verkhratsky, 2012).

**Figure 1:**
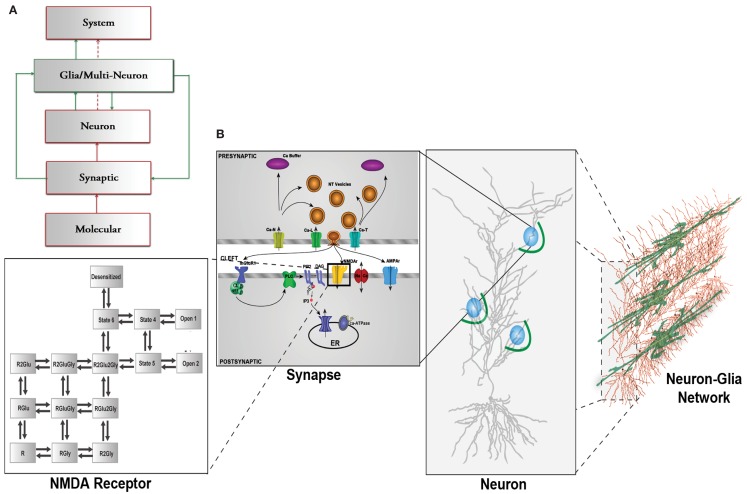
**(A)** CNS hierarchy with the traditional neuron-centric framework in red and the revised neuron-glia framework including glial interactions in green. **(B)** Multi-scale framework of the CNS hierarchy including molecular, synaptic, neuronal, and network level including glial cells., modified from Bouteiller et al. (2011) The molecular level is represented with kinetic schema. The synaptic level includes several molecular elements and their spatio-temporal interaction. The neuron level comprises morphologically realistic neuron model with synapses (blue circles) randomly located on dendritic branches surrounded by astrocyte processes (green arcs). The network level takes into account the interaction between neurons and glial cells.

In this paper, we present a computational modeling framework that spans across several hierarchical layers of the central nervous system (CNS), from the molecular to the synaptic, dendritic, and neuronal levels. The structure of this framework allows us to investigate the influence of glial cells at each of these levels and how they can modulate neuronal communication. Traditionally, high level brain processes are explained using the framework illustrated in red in Figure 1A where molecular dynamics at neuronal synapses are linked to system-level brain functions via synaptic neuronal signaling (Kandel et al., 1991). The new methodology we have chosen to explain and model these complex mechanisms is represented by the combination of the red, neural components, and the green, glial/astrocytic components, which have been added to account for the contribution and influence of astrocytes. Since glutamate is the most important neurotransmitter involved at excitatory synapses and in astrocytic signaling, we focus on modeling the effect of astrocytic glutamate transporters on neuronal spiking within this new framework (Figure 1A).

In the original, neuron-centric framework shown in red, synapses, and neurons are the fundamental building blocks of a network within which information exchange is mediated by molecular mechanisms. The lowest level of hierarchy in Figure 1A comprises elements that interact at the molecular level, such as the three classes of glutamate receptors, ionotropic receptor-channels, AMPAR (α-amino-3-hydroxy-5-methyl-4-isoxazolepropionic acid receptor) and NMDAR (*N*-Methyl-d-aspartate receptor), and mGluR (metabotropic glutamate receptor). At the synaptic level, ion fluxes through these receptor-channels give rise to synaptic responses of varying time courses and amplitudes depending on channel kinetics. These responses sum in a non-linear spatio-temporal manner and evoke spiking activity in neurons. A network of these neurons constitutes the system that emulates a physiological function.

The box outlined in green shows the inclusion of the previously underappreciated glial components (Nedergaard et al., 2003). One particular type of glial cell, the astrocyte, has thousands of processes and extensions that are often found in close proximity to hundreds of synapses, which it can potentially modulate (Halassa et al., 2007b).

Additionally, it has been discovered that astrocytes express large amounts of neurotransmitter receptors and transporters (Wang and Bordey, 2008). Since a neuron’s pre- and postsynaptic nerve terminals are often ensheathed by astrocytes, which participate in synaptic signal processing, the term tripartite synapse was coined (Araque et al., 1999).

Within a tripartite synapse, biochemical, and morphological studies suggest that excitatory amino-acid transporters (EAATs) expressed on astrocytes are of the type EAAT2. These glutamate transporters maintain low extracellular glutamate concentration, which prevents neurotoxicity in spinal cord, striatum, and hippocampus (Rothstein et al., 1996), and might play a functional role in regulating synaptic currents by clearing glutamate after its synaptic release (Bergles and Jahr, 1998; Diamond, 2005). Neuronal transporters (EAAT3) also take up glutamate from the extracellular space, however, at a significantly lower rate than astrocytes due to their lower expression levels (Rothstein et al., 1996). The role of neuronal transporters could be to limit glutamate spill-over and to slow down glutamate clearance by glial transporters (Diamond, 2001; Scimemi et al., 2009). The importance of astrocytes in the regulation of glutamate uptake, the transformation of glutamate to glutamine (Uwechue et al., 2012) for re-usage in synaptic transmission, and epilepsy pathogenesis has recently been reviewed (Coulter and Eid, 2012).

Several studies demonstrated that astrocytes not only uptake glutamate inside a tripartite synapse, but under certain conditions, can also release glutamate (Araque et al., 1998), through a process termed gliotransmission (Halassa et al., 2007a). This term describes the process of glutamate release from astrocytes due to an increase in intracellular calcium via mGluR-mediated mechanisms in response to neural signaling inside a tripartite synapse (Parpura et al., 1994; Fiacco and McCarthy, 2006; Zur Nieden and Deitmer, 2006).

Because of the astrocyte’ structural characteristics and biochemical signaling mechanisms, these cells may play an important role at the neuron and network levels of the CNS hierarchy (Volterra et al., 2002). However, the exact manner in which astrocytes communicate with neurons *in vivo* is still unclear. For example, the effect of glutamate uptake in synaptic transmission, and on neuronal spiking, is difficult to verify using state-of-the-art experimental procedures. Furthermore, several experimental and review articles have been published that challenge the tripartite synapse concept (Agulhon et al., 2010; Nedergaard and Verkhratsky, 2012), and raised a number of issue regarding the way astrocytes contribute to synaptic signaling by performing feedforward and/or feedback action through the uptake and release of neuro- and gliotransmitters (Smith, 2010).

One of the primary reasons why these disagreements cannot easily be resolved, is the lack of experimental techniques to directly study astrocytes and their biochemical signaling mechanisms (Nedergaard and Verkhratsky, 2012). Since astrocytes are generally not electrically excitable (do not generate action potentials), techniques for measuring glial cell activity mainly rely on imaging for *in vitro* studies or genetic manipulations for *in vivo* experiments. Hence, experiments are often performed under non-physiological conditions (Nedergaard and Verkhratsky, 2012), as *in vitro* experiments do not represent the natural environment of these cells and their responses might be drastically amplified. Disabling astrocytes through genetic manipulations is equally non-physiological and might result in compensatory effects *in vivo* (Smith, 2010).

One promising approach that can resolve these uncertainties is to use computational models. Such models provide opportunities to analyze the behavior of the system in response to various stimuli and conditions otherwise difficult to conduct experimentally, either due to the lack of the necessary technology, or difficulties in accessing readouts at the sub-cellular level.

In recent years, many computational modeling groups have demonstrated the glial influence within synaptic, neuronal, or network dynamics. Nadkarni and Jung (2007) have characterized astrocytic effects on spontaneous activity at the postsynaptic level. Silchenko and Tass (2008) have modeled the effects of glutamate release from astrocytes on neuronal depolarization and activity. Somjen et al. (2008) have used computer simulations to demonstrate that neuron-glia interactions are in part mediated by potassium ion fluxes between the two entities. Finally De Pitta et al. (2011) and Wade et al. (2011) have presented a modeling approach with bi-directional communication and synchrony between astrocytes and neuron clusters.

Our modeling approach integrates the dynamics from the molecular to the neuronal level and provides an original framework that allows for a better understanding of the effects of glia in a hierarchical manner. This modeling methodology mimics the structure of the CNS which spans several spatial and temporal scales and forms a hierarchical system from the bottom-up: molecular to synapse level, synapse to neuron level, and neuron to network level, with the inclusion of glial interactions. For example, the activation of receptors at single synapses, which are distributed along the dendritic tree of pyramidal neurons, sum in a non-linear fashion, which changes the membrane potential (Poirazi et al., 2003). These responses influence the network level, along with additional modulatory inputs from other pathways and inhibitory connections.

In this paper, we focus on the role of astrocytic glutamate uptake on synaptic responses and how it can modify neuronal spiking within the context of this modeling framework (Figures 1A,B). We observe that glial glutamate uptake is important to decrease desensitization of ionotropic glutamate receptors resulting in enhanced paired pulse facilitation for very short input intervals. Based on these observations, we propose that the time interval between input pulses and the interactions between receptors and transporters significantly contribute to neuron spiking patterns. Our model provides a unique method to assess critical parameters that can influence neural network behavior in relation to glutamate uptake.

## Methodology

The synaptic modeling platform EONS/Rhenoms™ (Elementary Objects of the Nervous System) was developed to configure detailed synapses and define the distribution and arrangement of molecular elements within a synaptic environment (Bouteiller et al., 2008). The models and their descriptions are available online at http://synapticmodeling.com/. Each of the individual synaptic elements are described by kinetic schema that characterize the behavior of receptors using parameters fitting the model’s responses to experimental data. The generic synapse model described here comprises presynaptic calcium buffers, voltage-dependent calcium channels, a single vesicular glutamate release site, glutamate diffusion in the synaptic cleft (Savtchenko and Rusakov, 2007), and binding of glutamate to postsynaptic ionotropic and metabotropic glutamate receptors (Ambert et al., 2010; Greget et al., 2011). Glutamate uptake is incorporated in the platform by including a model of a high-affinity glial glutamate transporter (EAAT2/GLT-1) adapted from Bergles et al. (2002), and a neuronal transporter (EAAT3) with parameters adapted from Larsson et al. (2004). Glutamate molecules released from presynaptic vesicles diffuse in the synaptic cleft. Glutamate molecules taken-up by these transporters are subtracted from those available at ionotropic and metabotropic receptors in the postsynaptic density. Numerous studies have quantified the tortuosity of the synaptic cleft and estimated the glutamate diffusion coefficient (Nielsen et al., 2004). The diffusion model used here has been adapted from Savtchenko and Rusakov (2007) to calculate glutamate concentration inside the cleft as a function of the distance of the receptor from the release site using Eq. 1. The glutamate diffusion coefficient is 0.4 μm^2^ ms^−1^. The total number of transmitter molecules released from the vesicle in the model is 3,000 per release event. The concentration of glutamate inside the cleft is determined by the following equation:

(1) $$\text{Glu}\left(r,t,Q,D,\delta \right)=\frac{Q}{4\pi \delta Dt}e^{\frac{-r^{z}}{4Dt}}$$

**Figure 2:**
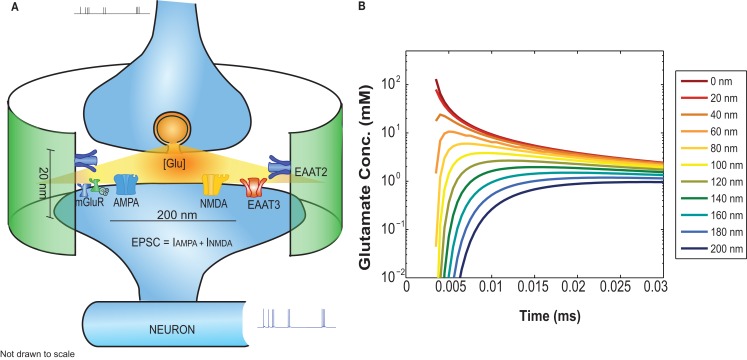
**(A)** Functional block diagram of the EONS synapse model including: glutamate diffusion inside the cleft, AMPAR, NMDAR, mGluR, and glutamate uptake mediated by glial (EAAT2) and neuronal transporters (EAAT3). The green cylindrical ensheathment represents the astroglial process on which EAAT2s are expressed. **(B)** The glutamate concentration profile as a function of time for receptors located at different distances relative to the glutamate release side.

Where Glu, *r*, and *D* represent the concentration of glutamate inside the cleft, the radial distance, and the diffusion coefficient, respectively. *Q* represents the number of glutamate molecules released instantaneously. “δ” stands for the height of the cleft and is maintained constant throughout the simulations at 20 nm. The glutamate profile seen by postsynaptic receptors is shown in Figure 2B.

**Figure A3:**
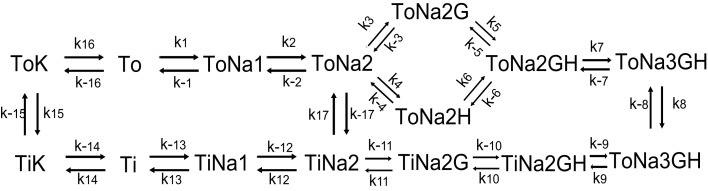
**Schematic of glutamate transporter receptor model with kinetic rate constants for glial glutamate transporter adapted from Bergles et al. (2002) and provided in the table below**.

The concentration levels of glutamate available at AMPAR and NMDAR is determined by their spatial location on the post synaptic density (PSD). The astrocytic glutamate uptake is calculated by the following rate equation for glutamate flux:

$$\begin{array}{llll} \frac{d\text{Gluo}}{dt} & =k_{6}\times \text{Na2ToH}\times \text{Glu}-k_{-6} & & \text{(2)}\\ & \times \text{Na2ToGH}+k_{3}\times \text{Glu}\times \text{Na2To}-k_{-3}\times \text{Na2ToG} & & \end{array}$$

where Na2ToH, Na2ToGH, Na2To, Na2ToG are the intermediate states that determine glutamate bound and transported by the transporter (see Figure A3 in Appendix). The values for parameters *k*_6_, *k*_3_ is 6 mM^−1^ ms^−1^ and for *k*_−6_, *k*_−3_, is 0.5 ms^−1^. All the other rate constants and ion concentrations are the same as reported in Bergles et al. (2002). The uptake rate in the equation above is for a single transporter channel. The diameter of the postsynaptic disk is 200 nm (Takumi et al., 1999) the height of the synaptic cleft is fixed at 20 nm (Savtchenko and Rusakov, 2007). The astrocyte membrane and its transporters are set at a distance of 400 nm from the release site. The distance of the transporter from the release site does not influence its uptake rate (Diamond, 2005). Transporters are expressed on astrocytes surrounding synapses with densities within a range of 6,500–13,000 μm^−2^ (Lehre and Danbolt, 1998; Diamond, 2005). The astrocyte is modeled as a cylindrical surface of height 20 nm and radius 400 nm as depicted by the green ensheathment around the synapse in Figure 2A. Assuming the maximum transporter density (13,000 μm^−2^) and a 50% astrocytic coverage surrounding the synapse, we calculate the number of glutamate transporters per synapse to be 650. For the same conditions but with 50% density, we calculate the number of transporters to be 325. Glutamate uptake from neuronal transporters is calculated in a similar manner to that by glial transporters (Eq. 2) with the kinetic rate constants adapted from Larsson et al. (2004) and a transporter density of 90 μm^−2^ (Holmseth et al., 2012). The total glutamate cleared from the receptor vicinity is obtained by multiplying the cleared glutamate rate *d*GluO*/dt*, integrating over the time steps of the simulation and multiplying by the number of transporters. This amount of cleared glutamate is then subtracted from the glutamate input concentration available at the receptors.

We used the AMPA receptor model described in detail in Robert and Howe (2003) which represents a 16 states model describing the receptor transitions between resting, desensitized, and conducting open states. Successive binding of two, three, and four glutamate molecules produces conformational changes leading to fast opening and closing of the channel.

The current through the channel is calculated by:

(3) $$I_{\text{AMPA}}=\text{n}{\text{b}}_{\text{AMPA}}\times \left(g_{2}O_{2}+g_{3}O_{3}+g_{4}O_{4}\right)\times \left(V-V_{\text{rev}}\right)$$

Where the open conducting states evolve as:

(4) $${Ȯ}_{i}=\left[M\left(\text{Glu}\right)\right]\cdot O_{i}$$

where *I*_AMPA_ is the current mediated by AMPA receptors, nb_AMPA_ is the number of AMPA receptors (in this study nb_AMPA_ is 80), consistent with reported AMPAR numbers between 46 and 147 at CA1 hippocampal synapses (Matsuzaki et al., 2001). *g*_2_, *g*_3_, *g*_4_ are unitary conductances with values 9, 15, and 21 pS associated with the channel in open states when 2, 3, and 4 glutamate molecules are bound respectively. The probabilities for the *O*_2_, *O*_3_, *O*_4_ states are calculated based on ODEs simulated using solvers in SBML™. The derivatives of open states ${Ȯ}_{i}$ (where *i* = 2, 3, 4) are calculated as a product of matrix *M* containing other states transition rate constants with input Glu and vector of currents states *O_i_*. *V*_rev_ is the reversal potential of the AMPAR (*V*_rev_ is 0 mV) and *V* is the membrane potential that changes dynamically during the simulation. More details on the model can be obtained from Robert and Howe (2003) and for kinetic rate parameters, please, see Section “Appendix.”

Glutamate concentration available at NMDAR after glutamate uptake is provided as input to the NMDAR model represented in a 15-state kinetic scheme, which includes agonist (glutamate) and co-agonist (glycine) binding sites, channel blockers (memantine and magnesium), as well as several antagonist sites. The kinetics of this model are borrowed from Ambert et al. (2010). For validation of the NMDAR model, various protocols were tested. For a single short pulse of glutamate, experimental results reported by Schorge et al. (2005) were used to validate the model. For long or repetitive glutamate inputs to the model, the kinetic parameters were adjusted to properly capture effects of desensitization and to match experimental data from Zhang et al. (2008). The equations to calculate NMDAR-mediated synaptic current are:

$$\begin{array}{rcll} I_{\text{NMDA}}=\text{n}{\text{b}}_{\text{NMDA}}\frac{I_{o}}{1+\left(\frac{Mg_{0}^{2+}}{K_{0}}\right){\text{e}}^{-\delta zF\Psi m∕RT}} & & & \text{(5)}\\ I_{o}=g\left(V-V_{\text{rev}}\right)O\left(t\right) & & & \\ g=g_{1}+\frac{g_{z}-g_{1}}{1+{\text{e}}^{\alpha \Psi m}} & & & \end{array}$$

where *I*_NMDA_ is the current mediated by NMDA receptors. nb_NMDA_ is set at 20 for this study consistent with observations made in Takumi et al. (1999) and Racca et al. (2000). *I*_o_ is the current associated with the open conducting state *O(t)* calculated using ODEs solved with kinetics described in Ambert et al. (2010). The magnesium concentration in the external solution is set to 1 mM; Ψ*m* is the electrical distance of the magnesium binding site from the outside of the membrane (set at 0.8); *R*, the molar gas constant (8.31434 J mol^-1^ K^-1^); *F*, the Faraday constant (9.64867.104 C mol^-1^); *T*, the absolute temperature (273.15 K); *g*_1_ and *g*_2_ are the conductances associated with the open states when one or two glutamate molecules are bound and are 40 and 247 pS respectively; α = 0.01 is the steepness of the voltage-dependent transition from *g*_1_ to *g*_2_.

The total synaptic current is calculated from the sum of AMPAR and NMDAR currents.

(6) $$I_{\text{syn}}=I_{\text{AMPA}}+I_{\text{NMDA}}$$

The synaptic currents calculated using Eq. (6) drive the neuron membrane potential and synapses are now approximately acting as current sources (Jaffe and Carnevale, 1999). A CA1 pyramidal cell model with active sodium and potassium channels and a morphology described in Jarsky et al. (2005) was used within the NEURON simulation environment (Hines and Carnevale, 1997). Synapses were placed at 16 random locations in stratum radiatum (middle one-third of the cell, 100–200 μm). The synaptic strength was tuned by a factor of 6 to reach threshold levels for neuronal spiking such that spiking probability was 1.

## Results

### Effect of astrocytic glutamate uptake on postsynaptic currents

The EONS/Rhenoms™ modeling platform allows the investigation of critical parameters that modulate synaptic transmission and neuronal spiking. In this paper we study the effects of EAATs within the astrocyte membranes surrounding CA1 hippocampal neurons. The result section begins with a demonstration of the model’s fidelity in replicating the influence of astrocytic glutamate uptake at the synapse level, as previously shown (Sarantis et al., 1993; Bergles and Jahr, 1998; Diamond, 2005). We then build on these results by investigating how astrocytic glutamate uptake influences ionotropic elements’ responses for different input stimulation protocols such as paired pulse and random interval trains (RITs). The molecular level effects, such as receptor desensitization and transporter saturation, arising due to the relative timing between the inputs (glutamate release from single vesicular sites) are demonstrated. We believe that understanding the interactions between these molecular level elements through simulation studies provide insights into synaptic level responses that are difficult to explore experimentally. In this work, we demonstrate how sub-cellular responses can affect neuronal spiking.

#### Astrocytic glutamate uptake decreases peak amplitudes of AMPAR-mediated EPSCs

AMPARs are known for their crucial role in mediating fast excitatory synaptic transmission. AMPARs and NMDARs co-exist at many central glutamatergic synapses (Bekkers and Stevens, 1989) and have very distinct kinetics contributing to the fast and slow components of the EPSCs respectively (Umemiya et al., 1999). It was previously shown that astrocytic glutamate transporters do not shape the decay of non-NMDA receptor-mediated synaptic responses (Sarantis et al., 1993).

**Figure 3:**
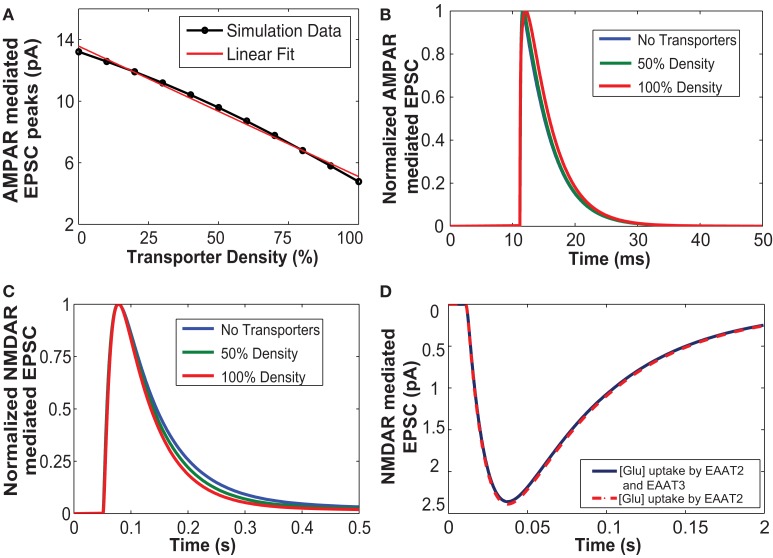
**(A)** The increased number of glutamate transporters affects the peak amplitude of AMPAR-mediated current due to uptake of glutamate. **(B)** Normalized responses of AMPA mediated EPSCs elicited from a single input pulse for cases with no transporters, 50% density and 100% density of astrocytic glutamate transporters. The decay time course of normalized AMPAR currents with 50% density and without any transporters did not show any change. **(C)** Glutamate uptake by the glial transporters affects the decay time course of NMDA receptor-mediated EPSC. An increase in the density of transporters results in an increase in the rate of uptake thus decreasing the time of decay of NMDA receptor-mediated EPSC. **(D)** NMDAR-mediated EPSCs with glial glutamate uptake (red), and with both glial and neuronal uptake (blue). The uptake mediated by neuronal transporters (EAAT3) is not significant.

We proposed to test if our model yielded the same effects and studied the role of glutamate uptake on current going through the AMPAR channels in response to a single vesicular release. We simulated AMPAR-mediated EPSCs for a single pulse input with 50, 100% glutamate transporter densities and without glutamate transporters. These three cases were chosen to demonstrate the effect of glutamate transporters at moderate and extreme cases of transporter density on astrocytes. Figure 3A shows the values of peak amplitude responses of AMPAR-mediated EPSCs plotted against varying density of glutamate transporters. AMPAR currents obtained here are in response to a single presynaptic input pulse eliciting a single vesicular release of glutamate as a function of the number of surrounding astrocytic glutamate transporters. Due to increased density of transporters and hence more glutamate uptake, glutamate input to AMPARs is decreased. As expected, the amplitude of AMPAR-mediated synaptic responses decreased with increase in the number of transporters. The red line in Figure 3A is a linear regression fit to the data (in black) with a correlation coefficient of *r*^2^ = 0.69. Figure 3B shows the normalized AMPAR-mediated EPSCs in response to a single release event with (50 and 100% density) and without transporters. These results indicate that the decay of the time course remains the same with 50% transporter density and without the transporters. However, at 100% density of transporters, the peak itself is shifted, which could be due to elevated glutamate uptake, but the time course decay is very similar. Since AMPARs are sensitive to glutamate concentration, removal of glutamate from the cleft due to buffering or uptake by EAATs modifies this concentration and thus affects the AMPAR-mediated responses.

#### Astrocytic glutamate uptake influences decay phase of NMDAR-mediated EPSC time course

Unlike for AMPARs, the responses of NMDARs are slower (Lester et al., 1990). Therefore, it is important to study the role of glutamate uptake on NMDARs. Numerous experimental studies report that glutamate uptake mediated by glia and neuronal transporters significantly influences the decay phase of NMDAR-mediated EPSCs (Bergles and Jahr, 1998; Bergles et al., 2002; Diamond, 2005).

Figure 3C shows the simulated responses of normalized NMDAR-mediated postsynaptic currents to a single release event with 50 and 100% densities, and without transporters. Our simulations confirm previously reported results suggesting that an increased number of EAATs leads to an increase in glutamate uptake, which causes a faster decay of NMDAR-mediated EPSC. Thus, increased expression and density of glutamate transporters could possibly account for the developmental changes that occur with age in NMDAR-mediated EPSCs between P14 and adult rat pyramidal cells (Diamond, 2005).

We also explored the role of the neuronal transporters, which are located within perisynaptic regions (He et al., 2000) on NMDAR-mediated EPSCs. Neuronal transporters at a density of 90  μm^−2^ (Holmseth et al., 2012) were placed within the presynaptic annulus of radius 100–400 nm. Figure 3D shows NMDAR-mediated EPSCs elicited in response to a single pulse taking into account glutamate uptake mediated by glial transporters (EAAT2) shown in red and the combined effect of glial and neuronal transporters (EAAT3) shown in blue. Our simulation results showed that uptake by neuronal transporters did not significantly affect EPSC responses, in contrast to glia-mediated glutamate uptake, which showed a stronger effect NMDAR-mediated EPSCs.

#### Astrocytic glutamate uptake effect on paired pulse responses is different for small and large input time intervals

**Figure 4:**
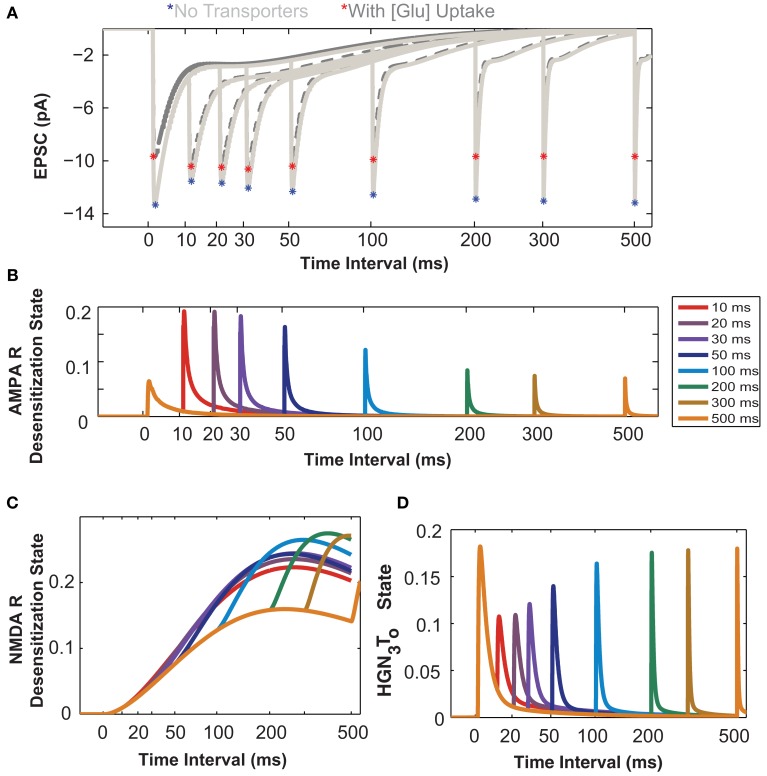
**Astrocytic glutamate uptake effect on paired pulse responses is distinct for small and large input time intervals**. **(A)** Composite EPSCs elicited by paired pulse stimulation plotted against input time intervals separated by 10–500 ms (Time axis in log scale to zoom into the effects at shorter input time intervals for all plots). Paired pulse depression (PPD) effect is observed for responses when no transporters are present (light gray, peaks marked by red asterisks). With the presence of transporters and astrocytic glutamate uptake (dark gray, peaks marked with blue asterisks), there is a paired pulse facilitation (PPF) effect observed for responses when the input time intervals are short. This reversal of effect from PPD to PPF is only apparent for shorter input time intervals. **(B)** The probability of the AMPARs in desensitization state as a function of input time intervals. These receptors are highly desensitized for shorter input time intervals. **(C)** The probability of NMDARs in desensitization state. The NMDARs are highly desensitized and this increases with increasing input time intervals. **(D)** HGN_3_T_o_ state probability of the glutamate transporter, when H^+^, Glu, 3 Na^+^ are bound to the transporter. The transporter recovers to this same state only after longer input time intervals (>200 ms).

In this section we investigate the influence of astrocytic glutamate uptake on EPSCs mediated by both AMPARs and NMDARs for a paired pulse input. The fast component of the EPSC is mediated by AMPARs and the slow component is mediated by NMDARs (Umemiya et al., 1999). The quantal value of EPSCs may vary with developmental stage or age of the animals (Bellingham et al., 1998), as differential expression and density of AMPA and NMDA receptors will result in different values of AMPAR- and NMDAR-mediated components. For this study, we use AMPA to NMDA receptor ratio of 80–20 (see Methodology). The paired pulse protocol is commonly used for testing presynaptic effects on EPSCs (Debanne et al., 1996) by changing the timing between two release events. Figure 4A shows the composite EPSCs to paired pulse inputs for different input intervals from 10 to 500 ms with (dark gray traces) and without glutamate transporters (light gray traces). The asterisks highlight the peak amplitudes of the EPSC with (red) and without (blue) glutamate uptake. To better demonstrate the effects of glutamate uptake for shorter input intervals, the results are plotted on a logarithmic time scale. In Figure 4A, the difference in the peak amplitude of the response to the first pulse with and without glutamate uptake is significant. For input intervals up to 100 ms, we observe paired pulse facilitation (peak amplitude of the second pulse is larger, as compared to that to the initial pulse) with glutamate transporters, and paired pulse depression (peak amplitude of the second pulse smaller, as compared to that to the initial pulse) when there are no transporters. For larger intervals, however, this facilitation/depression effect becomes less prominent in both cases, with and without glutamate transporters.

To better explain these effects, we take advantage of the modeling platform features to access the individual desensitization states (probability of receptors being desensitized) of AMPARs and NMDARs.

The probability of the desensitized state of AMPARs with two glutamate molecules bound is plotted as a function of paired pulse intervals in Figure 4B. At smaller input time intervals, the AMPAR enters into a desensitized state more rapidly, thus reducing its probability of being open or more responsive to the second pulse of glutamate, thereby decreasing the current through its channel. As shown in Figure 4C, the NMDA receptor also enters into a desensitized state and the probability of being in this state increases with increase in the time intervals between input pulses, as the receptor takes a much longer time to return to its original resting state. This desensitization property reduces the receptor’s ability to be in the open state in response to subsequent pulses for almost all interval ranges examined here.

Figure 4D shows the dynamics of the EAAT2 transporter in its HGN3T_0_ state (when glutamate, Na^+^, and H^+^ ions are bound and the transporter is facing outward). These results show that the glutamate transporter has to go through a recovery phase during which the clearance rate is slowed down. This indicates that the transporter may not be in its full capacity shortly after an initial pulse, thereby reducing its responsiveness to glutamate released on subsequent pulses. Supporting evidence for changes in glutamate transporter’s uptake rate and its effects have been reported for different input pulse intervals (Otis and Jahr, 1998; Diamond, 2005). In these studies, glutamate uptake rate was assessed through synaptically activated transporter–mediated anion currents (STCs).

In Figure 4A, the light gray traces show EPSCs responses elicited by paired pulse inputs. Blue asterisks mark the peak amplitude of the responses. The peak amplitudes on the second pulse elicited after intervals of 10 ms and up to 300 ms are much lower than the peak amplitude of the first pulse. This effect is known as paired pulse depression and it is due to the desensitization of the receptors during these input intervals, as described above. The peaks elicited by the second pulse slowly recover toward the first peak, as receptors return to the original responsive state. However, when glutamate uptake occurs, there is paired pulse facilitation, indicated by the red asterisks on the peaks of the dark gray traces for the shorter input intervals. This effect could be due to (i) a significant reduction in the peak amplitude of AMPARs because of glutamate uptake, as shown in Figure 3A for single pulse, as well as (ii) transporter’s recovery time to uptake glutamate with the same efficiency. As seen from these simulation studies, we hypothesize that inter-play between receptor desensitization and transporter recovery explains the clear differences in paired pulse responses in the presence (facilitation) or absence (depression) of astrocytic glutamate uptake. These differences disappear for longer intervals, as the transporter recovers to its initial state and receptors recover from desensitization. Thus, these simulation results highlight the importance of understanding the interactions between glutamate receptors and transporters at the molecular level. They also demonstrate the power of such computational modeling to facilitate understanding of these mechanisms.

### Astrocytic glutamate uptake influences neuronal spiking

The results presented in the previous sections demonstrate that glutamate uptake influences EPSC kinetics at the synaptic level. Given the hierarchical organization of the nervous system, a critical question we propose to address in this section is whether the local effects at the synaptic level can significantly affect neuronal signaling. The changes in input glutamate concentration profile due to EAATs affect ionotropic receptors and modify the kinetics of excitatory synaptic currents. These currents subsequently change membrane potential in dendrites and thus influence neuronal spiking activity. In order to effectively model these aforementioned phenomena, we simulated a neuron model using the NEURON simulation software that incorporate our detailed synaptic models, as described in the Section “Methodology.”

The neuron model used here (Jarsky et al., 2005) has a realistic CA1 pyramidal cell morphology with synapses distributed at random locations within 100–200 μm from the soma. The input to the system is a RIT (average rates at 2 and 5 Hz) to mimic low frequency spiking activities of the CA3 inputs to CA1 under physiological conditions. The synaptic input strength was chosen such that a spike was elicited by synchronous firing of all synapses. This condition makes the neuron very sensitive to threshold levels for firing. The neuron configuration described here is a specific case, and can be configured in multiple ways with respect to synaptic distributions and locations, strengths, and ion channel distribution. Previous work has shown how the rate of change of membrane potential contributes to neuronal firing of by modifying spiking threshold. In addition to these parameters, the synchrony of excitatory synaptic inputs and previous occurrence of an action potential can also determine the probability of occurrence of the next action potential (Azouz and Gray, 2000; Henze and Buzsaki, 2001).

**Figure 5:**
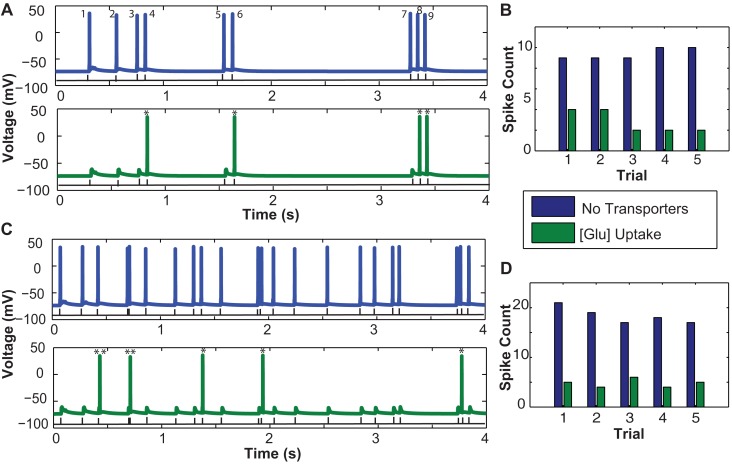
**Influence of astrocytic glutamate uptake on spiking activity of a CA1 pyramidal neurons at different input frequencies**. **(A)** Neuronal spiking activity elicited by a random input interval train with mean frequency of 2 Hz. The number of spikes occurring in the presence of glutamate uptake are much less (green) vs. when there are no transporters in the vicinity of synapses. **(B)** Number of spikes per trial within a span of 4 s without any transporters (blue) and with transporters and glutamate uptake (green). Across trials we observe a consistent decrease in the spike count. **(C)** Neuronal spiking activity elicited by a random input interval train with a mean frequency of 5 Hz. Similar effects of spike failure as seen in 2 Hz are observed. **(D)** Number of spikes per trial across five trials show the consistent failure of spikes due to increased glutamate uptake in the presence of transporters. Two critical events marked by * show that the spikes elicited without glutamate uptake (no transporters) and with glutamate uptake have a timing difference between 1 and 2 ms and ** indicates when timing lies in between 3 and 8 ms.

Figure 5A illustrates the spiking activity of a CA1 pyramidal neuron elicited by a 2-Hz RIT input in the absence (blue) or presence (green) of EAATs. The average number of spikes elicited in the presence of EAATs is always smaller, as compared to that in the absence of EAATs. Figure 5C shows the results for a 5-Hz RIT input. In addition to spiking activity, the Figure shows two critical events marked by * when spikes elicited without glutamate uptake and those with glutamate uptake have a timing difference between 1 and 2 ms and by ** when timing differences are between 3 and 8 ms. Spiking properties of neurons are dependent on multiple parameters; they could be due either to intrinsic neuronal properties, such as non-homogenous distribution of various ion channels, or to the diversity of synaptic inputs.

In Figure 5A, a RIT with mean frequency of 2 Hz containing nine pulses within a span of 4 s (black trace) is the input to the presynaptic terminals of all synapses. In the case without glutamate uptake or when no transporters were present, the neuron evoked nine output spikes at the soma, but only four spikes were elicited with glutamate uptake. Based on the paired pulse effects on EPSCs with and without glutamate transporters presented in Figure 4A, we expected to observe these effects on the number of spikes elicited at the neuron level. Between the two cases in Figure 5A, without (blue) and with (green) glutamate transporters, spike numbers 1, 2, 3, 5, and 7 failed to appear when there is glutamate uptake. Spike numbers 4, 6, 8, and 9 are not suppressed but arrive with a small delay. Looking at the time intervals between spikes 3 and 4, 5 and 6, and 8 and 9, which are all less than 200 ms, we can, see from the results in Figures 5A,C that the probability of a spike being suppressed due to glutamate uptake is more when the input inter-spike interval is longer than 200–300 ms. For longer inter-spike intervals, glutamate transporters should have recovered to their full potential for efficient uptake. Also, the subtle timing differences that occur between the two cases could be potentially due to the relative changes in NMDAR-mediated EPSC time course decay. The change in peak amplitude responses, mainly mediated by AMPARs, due to glutamate uptake may or may not drive the neuron’s membrane potential to spiking threshold values. Moreover it is the inter-play between the kinetics of AMPARs, NMDARs, and the transporters that can potentially lead to varying spiking patterns in neurons. We repeated a similar experiment, but with a higher input frequency of 5 Hz RITs (Figure 5C), which elicited between 19 and 22 spikes in the span of 4 s. Our results indicate that less spikes were elicited in the presence of glutamate uptake, in agreement with the observations with 2 Hz RIT responses. However, the spiking failure is comparatively less as there is a lower probability of spikes occurring with intervals longer than 200–300 ms. Five Hertz RIT contains inputs which are separated by intervals most likely shorter than 200 ms and all those paired pulse effects observed in Figure 4A at these intervals could possibly explain the spiking effects seen here.

To test the robustness of these simulations, several trials with RIT with the same mean frequency were run. It appears that the spike failure across trials elicited by 5 Hz was relatively consistent. However, as shown in Figure 5B, across trials elicited by 2 Hz RITs, there was more spike failure in some trials (such as 3 and 4). When we analyzed the simulation data, we observed that these trains had spikes separated by intervals longer than 300 ms. These interesting behaviors in spike failure may be attributed to the hypothesis described above, that the transporter would have recovered its uptake capacity thereby reducing amplitude and time course of AMPAR-mediated EPSCs.

These results are preliminary and were meant to demonstrate how simulation studies can be used to show that subtle changes in synaptic currents induced by glutamate uptake contribute to distinct neuronal spiking and temporal patterns. Changes in amplitude and time course of AMPAR- and NMDAR-mediated EPSCs, as shown in Figure 4A, were translated into subtle changes in spike arrival timings (Figures 5A,C) and spike failure (Figures 5B,D) with and without glutamate uptake. We built a model that could take into account receptor dynamics at elaborate synapses. Some of these dynamics, such as time course decay and amplitudes, were influenced by glutamate uptake mediated by glutamate transporters present on the astrocytic membrane surrounding these synapses. These results thus show the relevance of astrocytic mediated glutamate flux interactions between synapses and thus their effects on neurons.

## Discussion

In this paper we focused to study the role of glutamate uptake mediated by glutamate transporters present on astrocytic membranes surrounding CA1 hippocampal synapses on synaptic transmission and neuronal spiking. First we showed that the model was able to reproduce previously observed phenomena regarding the role of glutamate uptake on synaptic transmission by modulating responses mediated by AMPA and NMDA receptors. This was achieved by removing glutamate molecules that are bound to or transported by glutamate transporters from those available at the levels of the receptors. Previous studies have emphasized the importance of taking into account both glutamate diffusion and binding to transporters to determine changes in the decay kinetics of synaptic glutamate concentration (Wadiche et al., 1995). In agreement with this study, glutamate is removed from synaptic cleft by diffusion, and binding to and transport by glutamate transporters.

Some of the phenomena demonstrated here under the conditions we used are summarized:

(i) An increase in the density of astrocytic glutamate transporters results in a decrease in AMPAR-mediated EPSC’s peak amplitude. This effect is due to the rapid decrease in glutamate concentration mediated by the transporters. However, for a certain density of transporters and under the assumption that there is 50% ensheathment surrounding these synapses, the time course of AMPAR-mediated EPSCs is not influenced, as previously reported (Sarantis et al., 1993). Some experimental and simulation studies however show that there is no effect on AMPAR peak amplitudes (Zheng et al., 2008). This lack of effect could be due to a much higher density of transporters as compared to receptors and also to Monte Carlo diffusion studies that assumed a different configuration of glutamate diffusion, receptors kinetics, and transporter arrangement.
(ii) Astrocytic glutamate uptake has a more predominant effect on the decay phase of NMDAR-mediated EPSCs. This result is consistent with previously reported experimental results (Diamond, 2005). Our simulations allowed us to closely examine the effect of glutamate uptake on the desensitization properties of these receptors as well.
(iii) These two effects on amplitude and time course on EPSCs, combined with the transporter’s recovery behavior following paired pulse stimuli separated by short time intervals give rise to interesting dynamics. When glutamate uptake is not considered, there is paired pulse depression for responses to stimuli delivered at short intervals. However in the presence of glutamate uptake, paired pulse facilitation is observed. The transporter’s recovery kinetics are often neglected based on the assumption that, at physiological temperature, they have large capacity and respond to high frequency stimuli in a similar way (Wadiche and Kavanaugh, 1998; Auger and Attwell, 2000). However, modifying their kinetics as a function of temperature and pH may give rise to different outcomes. The importance of neuronal transporters was shown in studies where reduced expression of neuronal transporters (EAAT3) can lead to behavioral abnormalities (Sepkuty et al., 2002). In the current work, we included the EAAT3 kinetic model described in Larsson et al. (2004). EAAT3 type transporters are localized at dendrites and soma, and especially at perisynaptic regions (He et al., 2000). However, their role in mediating glutamate uptake is debated because of their low expression density, 1%, as compared to other types of EAATs expressed mainly by glia with densities of 20% for GLAST and 80% for GLT-1 (Holmseth et al., 2012). Experimental and simulation studies by Scimemi et al. (2009) show that neuronal transporters may slow down glutamate clearance time by astrocyte transporters and that they can influence NMDAR-mediated synaptic transmission. Glutamate molecules bound to efficient neuronal transporters are more likely to be transported once bound, than to be unbound (Otis and Jahr, 1998). Studies using knock out models of EAAC1/EAAT3 showed no significant changes in AMPAR- and NMDAR-mediated EPSCs for single vesicle release (Scimemi et al., 2009), which was the basic assumption of the synapse model used here (see Methodology). From other simulation studies by Diamond (2001) it is hypothesized that neuronal transporters might influence perisynaptic NMDA receptors, as they are more likely to be activated by glutamate spill-over from neighboring synapses. We tested the influence of neuronal transporters on NMDAR-mediated EPSCs at 90 μm^−2^ density and found that they had no insignificant effect on EPSC profile. Higher densities of neuronal transporters might induce a greater influence on EPSCs, but given the experimental findings mentioned above, it is generally accepted that they exhibit a low density of expression. Since the effects contributed by neuronal transporters were negligible, we focused in this study on understanding the changes in neuron spiking behavior induced by glutamate uptake mediated by astrocytic transporters. This focus was also a modeling choice to reduce the computational load in simulating synapses with too many elements. Exploring the influence of neuronal transporters with different kinetic parameters and density may or may not have an effect, as previously discussed and explored in previous studies.
(iv) All the above effects at the molecular and synaptic levels are translated into conductance changes with varying amplitudes and time courses that impact the temporal coding and spiking of neurons. Blocking glial glutamate uptake may have serious consequences on raising glutamate concentration to neurotoxic levels and causing epileptic conditions (Rothstein et al., 1996). These effects have been shown in both experimental and simulation studies (Oyehaug et al., 2012). In our simulations, we examined the effects of glutamate uptake on neuron spiking behavior elicited by 2 and 5 Hz RIT stimuli. Neurons with blocked astrocytic glutamate uptake showed higher spike counts. We attribute the failure of spikes in the presence of glutamate uptake mainly to the reduced levels of glutamate (i) that decrease synaptic amplitudes mediated by AMPARs and (ii) time course decay mediated by NMDARs, which also cause subtle differences in spike arrival. A closer look at the pattern of spike generation between the two cases without and with glutamate uptake shows that, spike usually occurs even after glutamate uptake, when the timing between the input pulses is less than 200–300 ms, implying that sometimes, the spike could be evoked because the transporters have not cleared glutamate levels up to optimal levels required for suppressing spike generation. Note that in this model the strength and number of synapses were chosen such that the neuron membrane potential reached threshold values easily. These parameters were selected in order to test the influence of glutamate uptake on spike activity around the membrane potential threshold for spike generation. Under our assumed conditions, the simulation studies show that glutamate uptake mediated by astrocytic transporters have a significant impact on neuronal spiking. To test the robustness of these results we ran several trials and found that the spike failure rates were more predominant for 2 Hz RITs, because there seems to be a higher probability of input trains with inter-spike intervals separated by more than 500 ms, when transporters and receptors have completely recovered for efficient glutamate uptake and responses, respectively.

We also underscore the diversity of synapses that arises from the variability in spatial location of the receptors, which we are investigating in a separate study. It is interesting to note that most of the receptors, such as NMDARs and mGluRs, have many modulatory sites, which consequently increase the number of parameters needed in the simulation. Our highly configurable geometric synapse model allows for the exploration of various parameters that influence sub-cellular/molecular level interactions and their direct or indirect influence on the synapse and neuron levels. By linking such complex unified model of a synapse to morphologically realistic models of neuron within the NEURON simulation environment, we can investigate this complexity in an orderly and hierarchical fashion. This modeling effort allows for the investigation of key phenomena that are otherwise difficult to explore through mainstream reductionist modeling approaches. The main technical drawback of this kind of approach is the computational overhead involved. The modeling paradigm itself is complex in its nature due to (i) the level of parametric details and (ii) the time scale of processing of some elementary models, which in some cases takes place within tenths of microseconds, thereby slowing down the entire system.

The astrocyte model presented here is not a complete model, and astrocytes are known for their role in influencing synaptic transmission beyond glutamate uptake and clearance. This model needs to be expanded to incorporate other important features, such as direct neurotransmitter release from astrocytes and signaling to neurons. All synapses are wrapped differently by astrocyte processes, covering smaller or larger areas with different levels of transporter expression.

We have assumed for simplicity a constant wrapping for each individual synapses, which may not be necessarily true *in vivo*. Parametric models, where the model behavior is explained by a set of parameters are in general limited by the scope of available experimental evidence. However, with a parametric modeling paradigm, we can test the reliability and sensitivity of these parameters. The observations described through our simulation studies are still preliminary and the modeling architecture established here will enable us to further investigate the effects caused by changing the amount of ensheathment around the synapses, as the density of EAATs in both glia and neurons appears to play an influential role in shaping synaptic glutamate concentration profile and its functional consequences. Including other details of a tortuous path for glutamate and including extrasynaptic NMDARs, may also affect synaptic responses. Future modeling efforts will be directed toward investigating the hierarchical effects of astrocytes on sub-populations of neurons and synapses contacted by astrocyte processes, by incorporating geometry-related considerations. Here, we demonstrated the hierarchical link between synaptic currents to spike generation while also taking into account astrocytic glutamate uptake effects on molecular elements. This is a novel approach and one of the few times such a link has been shown. These results may have significant implications for understanding glial cell effects on nerve cell membrane potential and thus, nerve cell spiking, i.e., neuronal information flow. These results also are important because they strongly suggest that glial cell uptake of synaptic glutamate during neuron-to-neuron synaptic transmission should influence spike-dependent processes that are relevant to secondary messenger pathways and other long-term effects. This comprehensive framework will allow investigating complex mechanisms within large neuron/glia networks, including neurodegenerative diseases and their underlying processes. We can identify the most sensitive parameters in neuron glial interactions and develop different testing paradigms to understand the molecular basis of diseases associated with astrocytic dysfunction.

## Conflict of Interest Statement

Jean-Marie C. Bouteiller, Michel Baudry and Theodore W. Berger have a conflict of interest. The University of Southern California holds an equity interest in Rhenovia Pharma and also has received licensing income from Rhenovia Pharma.

## Figures and Tables

**Figure A1:**
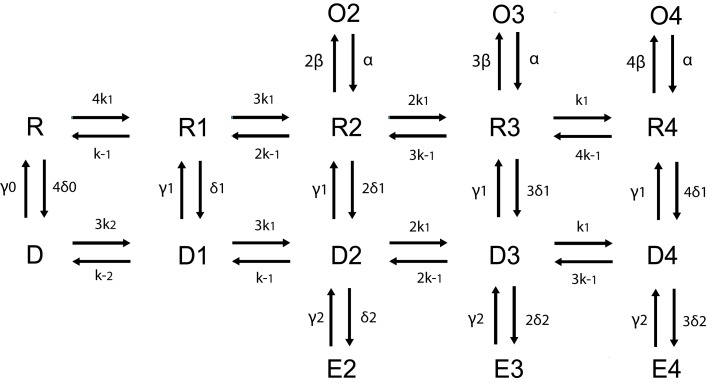
**Schematic of 16 states AMPA receptor model adapted from Robert and Howe (2003) with kinetic rate constants provided in the table below**.

**Figure A2:**
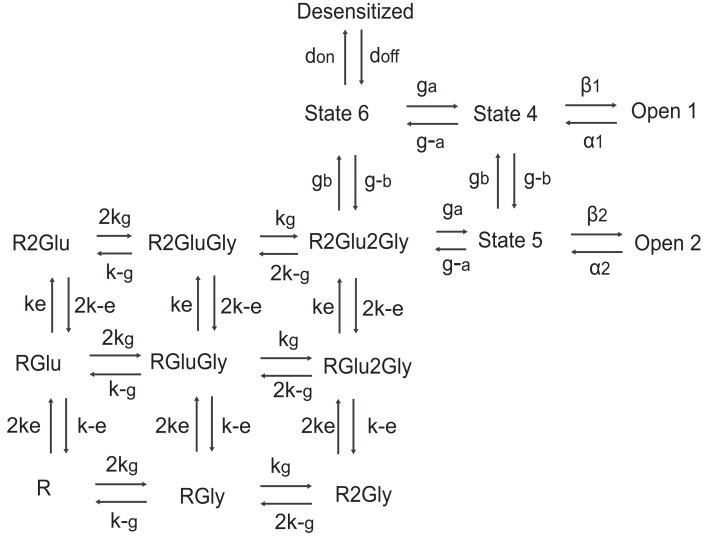
**Schematic of 15 states NMDA receptor model with kinetic rate constants adapted from Schorge et al. (2005) provided in the table below**.

## Appendix

EONS (Elementary Objects of the Nervous System) synaptic modeling platform is available online at http://synapticmodeling.com

The user guide is described here: http://www.synapticmodeling.com/eons_v1_user_guide.html

This software is implemented using JAVA. A library of elementary models is available within this platform.

Each elementary model within this is developed using Cell Designer™ platform. After testing with several pulse protocols, we integrated these into the EONS software, where elementary models interact with each other, i.e., one model’s output represents an input to another model.

The elementary model represents the receptor behavior in various intermediate states such as resting to inactive to open states. The time-dependent evolution of the states are calculated using ODEs and the number of equations depends on the number of states modeled.

Ẋ=M.X

**Table:**

Model	Parameters	Descriptions	Values
Glutamate diffusion: $\text{Glu}\left(r,t,Q,D,\delta \right)=\frac{Q}{4\pi \delta Dt}e^{\frac{-r^{z}}{4Dt}}$	*Q*	Number of glutamate molecules	3,000
	δ	Cleft height	20 nm
	*D*	Glutamate diffusion coefficient	0.4 μm^2^ ms^−1^
	*R*	Distance between the release site and the receptors or transporters	0–400 nm
	*t*	Time	ms
Glutamate uptake rate per transporter: $\frac{d\text{Gluo}}{dt}=k_{6}\times \text{Na2ToH}\times \text{Glu}-k_{-6}\times \text{Na2ToGH}+k_{3}\times \text{Glu}\times \text{Na2To}-k_{-3}\times \text{Na2ToG}$	*k*_6_, *k*_3_ *k*_−6_, *k*_−3_	Forward rate constants Backward rate constants	6 mM^−1^ ms^−1^ 0.5 ms^−1^
Glutamate at receptor after uptake: Glu – ∈t*d*Gluo	N2ToH, N2ToGH, N2To, N2ToG	Intermediate states of the glutamate transporter	Dynamically change during simulation
		Number of glial glutamate transporters	325
		Number of neuronal glutamate transporters	42
AMPAR-mediated current: $I_{\text{AMPA}}=\text{n}{\text{b}}_{\text{AMPA}}\times \left(g_{2}{\text{O}}_{2}+g_{3}{\text{O}}_{3}+g_{4}{\text{O}}_{4}\right)\times \left(V-V_{\text{rev}}\right)$ Robert and Howe (2003)	nb_AMPA_	Number of AMPA receptors	80
	*g*_2_, *g*_3_, *g*_4_	Unitary channel conductances associated with open states *O*_2_, *O*_3_, *O*_4_ updated during simulation	9,15, 21 pS respectively
	*V*	Membrane potential	Dynamically changes during simulation
	*V*_rev_	Reversal potential	0 mV
NMDAR-mediated current: $\begin{array}{l} I_{\text{NMDA}}={\text{nb}}_{\text{NMDA}}\frac{Io}{1+\left(\frac{Mg_{0}^{2+}}{K_{0}}\right){\text{e}}^{-\delta zF\Psi m/RT}}\\ I_{o}=g(V-V_{\text{rev}})\;O\;(t)\\ g=g_{1}+\frac{g_{z}-g_{1}}{1+e^{\alpha \Psi m}} \end{array}$	*nb*_NMDA_	Number of NMDA receptors	20
	*IO*	current associated with the open conducting state *O*	Dynamically changes during simulation
	*V*	Membrane potential	Dynamically changes during simulation
	*V*_rev_	Reversal potential	0.7 mV
	*Mg*^2+^	External Mg concentration	1 mM
	α	is the steepness of the voltage-dependent transition from *g*1 to *g*2	0.01
	*R*	molar gas constant	8.31434 J mol^-1^ K^-1^
	*F*	Faraday constant	9.64867.104 C mol^-1^
	*T*	absolute temperature	273.15^^^ K
Synaptic current: *I*_syn_ = *I*_AMPA_ + *I*_NMDA_	*I*_AMPA_	Current through AMPARs	Dynamically changes during simulation
	*I*_NMDA_	Current through NMDARs	Dynamically changes during simulation
Neuron model please, see Jarsky et al. (2005)	Simulation archive available from http://dendrites.esam.northwestern.edu/		

### Additional rate constants

#### AMPAR model

**Table:**

Parameter	Values
*k*_1_	10 mM^−1^ ms^−1^
*k*^−1^	7 ms^−1^
*k*_2_	10 mM^−1^ ms^−1^
*k*^−2^	4.1e^−4^ ms^−1^
γ_o_	0.001 ms^−1^
δ_o_	3.3e^−6^ ms^−1^
γ_1_	0.42 ms^−1^
δ_1_	0.017 ms^−1^
γ_2_	0.2 ms^−1^
δ_2_	0.035 ms^−1^
β	0.55 ms^−1^
α	0.3 ms^−1^

#### NMDAR model

**Table:**

Parameter	Values
*k*_e_	8.3 Mm^−1^ ms^−1^
*k*_−e_	0.0263 ms^−1^
*k*_g_	10 mM^−1^ ms^−1^
*k*_−g_	0.0291 ms^−1^
*g*_b_	0.0671 ms^−1^
*g*_−b_	0.15 ms^−1^
*g*_a_	2.03 ms^−1^
*g*_−a_	22.8 ms^−1^
β_1_	35.2 ms^−1^
α_1_	0.728 ms^−1^
β_2_	0.787 ms^−1^
α_2_	11.2 ms^−1^
*d*_on_	0.03 ms^−1^
*d*_off_	9.5e^−4^ ms^−1^

#### Glial transporters

**Table:**

Parameter	Values
*k*_1_; *k*_−1_	0.01 mM^−1^ ms^−1^; 0.1 ms^−1^
*k*_2_; *k*_−2_	0.01 mM^−1^ ms^−1^; 0.5 ms^−1^
*k*_3_; *k*_−3_	6 mM^−1^ ms^−1^; 0.5 ms^−1^
*k*_4_; *k*_−4_	60,000 mM^−1^ ms^−1^; 0.7 ms^−1^
*k*_5_; *k*_−5_	60,000 mM^−1^ ms^−1^; 0.7 ms^−1^
*k*_6_; *k*_−6_	6 mM^−1^ ms^−1^; 0.5 ms^−1^
*k*_7_; *k*_−7_	0.01 mM^−1^ ms^−1^; 1 ms^−1^
*k*_8_; *k*_−8_	2 ms^−1^; 1.9 ms^−1^
*k*_9_; *k*_−9_	1 ms^−1^; 0.04 mM^−1^ ms^−1^
*k*_10_; *k*_−10_	3 ms^−1^; 90,000 mM^−1^ ms^−1^
*k*_11_; *k*_−11_	3 ms^−1^; 0.1 mM^−1^ ms^−1^
*k*_12_; *k*_−12_	100 ms^−1^; 20 mM^−1^ ms^−1^
*k*_13_; *k*_−13_	100 ms^−1^; 100 mM^−1^ ms^−1^
*k*_14_; *k*_−14_	1 mM^−1^; 1 ms^−1^
*k*_15_; *k*_−15_	0.04 ms^−1^; 0.01 ms^−1^
*k*_16_; *k*_−16_	20 ms^−1^; 1 mM^−1^
*k*_17_; *k*_−17_	0.0014 ms^−1^; 1.0e^−5^ ms^−1^

#### Neuronal transporters

**Table:**

Parameter	Values
*k*_1_; *k*_−1_	0.01 mM^−1^ ms^−1^; 2.5 ms^−1^
*k*_2_; *k*_−2_	0.01 mM^−1^ ms^−1^; 2.5 ms^−1^
*k*_3_; *k*_−3_	6.8 mM^−1^ ms^−1^; 0.3 ms^−1^
*k*_4_; *k*_−4_	60,000 mM^−1^ ms^−1^; 0.7 ms^−1^
*k*_5_; *k*_−5_	60,000 mM^−1^ ms^−1^; 0.7 ms^−1^
*k*_6_; *k*_−6_	6 mM^−1^ ms^−1^; 0.5 ms^−1^
*k*_7_; *k*_−7_	0.01 mM^−1^ ms^−1^; 1 ms^−1^
*k*_8_; *k*_−8_	0.5 ms^−1^; 0.55 ms^−1^
*k*_9_; *k*_−9_	0.8 ms^−1^; 0.04 mM^−1^ ms^−1^
*k*_10_; *k*_−10_	6 ms^−1^; 90,000 mM^−1^ ms^−1^
*k*_11_; *k*_−11_	3 ms^−1^; 1 Mm^−1^ ms^−1^
*k*_12_; *k*_−12_	0.5 ms^−1^; 2 mM^−1^ ms^−1^
*k*_13_; *k*_−13_	4 ms^−1^; 1 mM^−1^ ms^−1^
*k*_14_; *k*_−14_	0.1 mM^−1^ ms^−1^; 10 ms^−1^
*k*_15_; *k*_−15_	0.05 ms^−1^; 0.005 ms^−1^
*k*_16_; *k*_−16_	0.8 ms^−1^; 0.01 mM^−1^ ms^−1^
*k*_17_; *k*_−17_	0.008 ms^−1^; 1.0 e^−5^ ms^−1^

Comparison between results showing NMDAR-mediated EPSCs simulated in EONS synaptic modeling platform. The blue trace represents the normalized EPSC waveform when there are no transporters. In the presence of glutamate transporters and due to glutamate uptake, we observed the decay phase shift inward indicating a faster decay time (red trace). This is qualitatively compared to the experimental findings (shown in inset) from Diamond (2005). The normalized NMDAR-mediated EPSCs shown in the inset, control condition (black trace), and with the addition of TBOA which blocks the transporter’s uptake activity. This is shown in gray trace. Note that with transporters and increased glutamate uptake, the decay phase of the time course decreases.
